# A Case of Myxedema Coma and Respiratory Failure

**DOI:** 10.7759/cureus.43747

**Published:** 2023-08-19

**Authors:** Ashley Clarke

**Affiliations:** 1 Internal Medicine, Ocean University Medical Center, Brick Township, USA

**Keywords:** hypercapnia, myxedema, hypothyroidism, myxedema coma, respiratory failure

## Abstract

Myxedema coma is a rare, but potentially fatal condition due to severe hypothyroidism, and most commonly seen in patients with long standing, untreated hypothyroidism. Here, I report a case of a 75-year-old male who presented to the emergency department with acute respiratory failure and altered mental status. Interestingly, this case led to a new diagnosis of hypothyroidism presenting in its most severe form - myxedema coma. This case highlights the diagnostic challenges in identifying patients with myxedema coma and recognizing its potential role as a cause of respiratory failure.

## Introduction

Thyroxine (T4) and its more active form, triiodothyronine (T3), are the primary thyroid hormones responsible for the body's metabolism, and cardiovascular, neurologic, and muscular function. Hypothyroidism is due to underproduction or impaired release of these hormones. Fortunately, due to widely available thyroid stimulating hormone (TSH) assay testing, primary hypothyroidism has become easily detected and treated, leaving complications such as myxedema coma a rare presentation. 

Myxedema coma is most commonly seen in older individuals, particularly women, with prior history of hypothyroidism, neck surgery, or radioiodine therapy. Classic clinical features include hypothermia, bradycardia, altered mental status, respiratory depression, and other signs of multi-organ dysfunction. These symptoms and signs are often non-specific or attributed to other underlying illnesses, particularly in the initial stages of stabilization. As presented in this index case, we see a unique presentation of myxedema coma- acute respiratory failure.

## Case presentation

**Figure 1:**
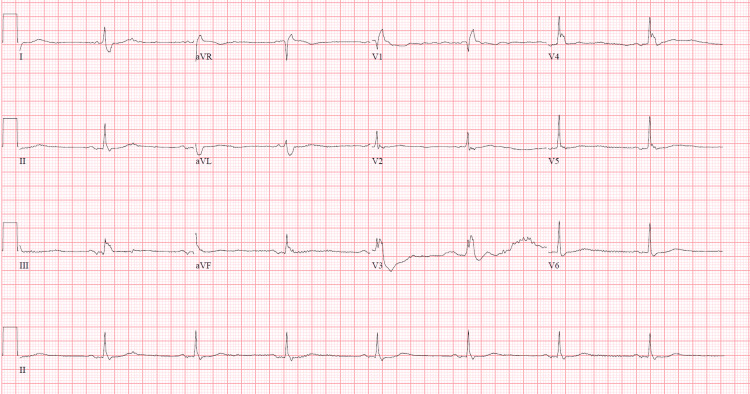
EKG showing sinus bradycardia and right bundle branch block EKG - electrocardiography

This is a 75-year-old male with a past medical history of chronic obstructive pulmonary disease (COPD), atrial fibrillation, and a one-year history of cognitive decline, who presented to the emergency department with worsening dyspnea and altered mental status. On presentation, he had hypotension responsive to intravenous fluids, pulse 47, and SpO2 84% on room air which normalized on oxygen via non-rebreather. On examination, he was lethargic and disoriented with the respiratory exam revealing prolonged expiration and decreased breath sounds. Facial puffiness and non-pitting edema of the extremities were also present, but there was no evidence of neck masses or bruits. Laboratory data was significant for white cell count 12.8 x 109/L, blood urea nitrogen 35 mg/dL, and creatinine 1.67 mg/dL (Table 1). Arterial blood gas showed respiratory acidosis with pH 7.22, pCO2 95.8 (Table 2). Blood and urine cultures were taken. The urinalysis and chest X-ray done were unremarkable; however, the electrocardiogram revealed sinus bradycardia with an old right bundle branch block (Figure 1).

**Table 1:** Laboratory data from the day of admission

Lab value	Result	Reference range
Hemoglobin	11.6	12.0 - 17.5 g/dL
Hematocrit	38.4	36 - 53%
White blood cell	12.8	4.5 - 11.0 x 10^3^/uL
Platelet	264	140 - 450 1 x 10^3^/uL
D-dimer	686	<500 ng/mL
Sodium	140	136 - 145 mmol/L
Potassium	4.3	3.5 - 5.2 mmol/L
Chloride	95	96 - 110 mmol/L
Carbon dioxide	36	24 - 31 mmol/L
Blood urea nitrogen	35	5 - 25 mg/dL
Albumin	3.3	3.5 - 5.0 g/dL
Creatinine	1.67	0.61 - 1.24 mg/dL
Calcium	8.6	8.5 - 10.5 mg/dL
Procalcitonin	<0.05	<0.05 ng/mL
Lactic acid	1	0.5 - 1.9 mmol/L

**Table 2:** ABG on admission ABG - arterial blood gas, pH - glood pH, PaO2 - arterial oxygen partial pressure, PaCO2 - arterial carbon dioxide partial pressure, HCO3 - bicarbonate ion concentration, SaO2 - arterial oxygen saturation, FiO2 - fraction of inspired oxygen, O2 - oxygen

Parameters	Results	Normal range
pH	7.227	7.35 - 7.45
PaO2	413.3	75 - 100 mmHg
PaCO2	95.8	35 - 45 mmHg
HCO3	38.9	22 - 28 mEq/L
SaO2	99%	>95%
FiO2	1	Varies based on therapy
O2 therapy	Non-rebreather mask	-

He was started on IV steroids, nebulizers, and empiric antibiotics and placed on bilevel-positive airway pressure ventilation (BIPAP) for treatment of COPD exacerbation and possible sepsis. After being stabilized, he was transferred to the intensive care unit (ICU) for close monitoring. In the ICU, he remained bradycardic and developed hypothermia and worsening hypotension requiring inotropic support. Given there was no identifiable source of infection, with a normal lactate level and normal procalcitonin, additional workup was done, which revealed significantly elevated TSH 82.1 uIU/mL (normal: 0.3 - 4.5 uIU/mL), T4 <0.25 ng/dL (normal: 0.50 - 1.26 ng/dL), and serum cortisol 19.41 mcg/dL (normal: 3.44 - 16.76 ug/dL). On further review, he had no prior history of thyroid dysfunction, radioiodine therapy, or ingestion of medications that disrupt thyroid function. This sparked a new differential diagnosis of severe decompensated hypothyroidism. In fact, the myxedema score [1] was calculated to be 80, highly suggestive of myxedema coma. He was subsequently given IV levothyroxine 200 mcg, IV hydrocortisone 100 mg, and continued on vasopressors with supportive care. 

Despite therapy and resolution of the hypercapnia, the patient became more altered and required intubation for airway protection. He was transferred to a tertiary hospital the following day for further management, where he was continued on IV Levothyroxine 75mcg daily, which led to normalization of T4 levels by day seven of treatment. Further investigations done to determine the underlying etiology of hypothyroidism were negative for thyroglobulin and thyroid peroxidase antibodies. He was successfully extubated and transitioned to oral levothyroxine 125 mcg and an oral prednisone taper. On day 15 of admission, he was deemed fit for discharge with close endocrinology follow-up. Unfortunately, the patient had a decline in functional status leading to two readmissions within one month of discharge from the hospital. Due to his overall poor prognosis, he was transitioned to hospice care and later expired. 

## Discussion

Myxedema coma is a misnomer for severe hypothyroidism, which typically presents as altered mentation instead of coma. The incidence of myxedema coma is not well studied in the United States; however, a European study estimated an incidence of approximately 0.22 cases per 100,000 per year in the Western world [2]. It is most commonly seen in elderly patients with long-standing, untreated, or undertreated hypothyroidism. This severe form of hypothyroidism is often associated with an acute infection; however, other precipitating factors may be found. These include non-adherence with thyroid hormone replacement therapy, stroke, heart failure, gastrointestinal bleeding, metabolic disturbances, or medications such as anesthetics, amiodarone, or lithium which affect thyroid hormone synthesis [2, 3]. These precipitating factors disrupt the neurovascular adaptations the body has made to achieve homeostasis resulting in multiorgan dysfunction. 

Clinically, in addition to altered mental status, other common presenting symptoms include lethargy, confusion, generalized edema, dry hair and skin, voice changes, bradycardia (which ranges from sinus bradycardia, bundle branch block or complete heart block), respiratory depression, and delayed deep tendon reflexes. Lab abnormalities typically include anemia, elevated creatinine, elevated transaminases, hyperlipidemia, and evidence of hypoventilation [4]. The hallmark of hypothyroidism is an elevated TSH and low T3 and T4, which may result several hours after testing and delay the confirmation of thyroid dysfunction.

In this 75-year-old male, his initial presentation of altered mental status was attributed to CO2 narcosis from COPD exacerbation, while hypothermia, leukocytosis, and altered mental status were also attributed to sepsis. However, due to the patient's bradycardia, there was a suspicion for hypothyroidism, for which a TSH level was ordered, indicating a new diagnosis of hypothyroidism. Given the diagnostic challenges, possible delay in laboratory values, and urgency in early treatment of myxedema coma, a clinical tool was developed in 2014 to aid in early recognition and treatment. This tool predominantly focuses on clinical features of multiorgan dysfunction (Tables 3, 4).

**Table 3:** Scoring system for myxedema coma Temp - temperature in °F, CNS - central nervous system, GI - gastrointestinal, CVS - cardiovascular, HR - heart rate, EKG - electrocardiogram * indicates features present in the current case

Clinical feature	Points
Thermoregulation	
Temp >95	0
Temp 89.6 - 95	10*
Temp <89.6	20
CNS effects	
Absent	0
Somnolent	10*
Obtunded	15
Stupor	20
Coma/ seizures	30
GI effects	
Anorexia/ abdominal pain/ constipation	5
Decreased intestinal motility	15
Paralytic ileus	20
Precipitating event	
Absent	0
Present	10
CVS effects	
HR >60	0
HR 50-59	10
HR 40-49	20*
HR <40	30
Other EKG changes (QT prolongation, low voltage, bundle branch block, heart blocks, non-specific ST-T changes )	10
Pericardial/pleural effusion	10
Pulmonary edema	15
Cardiomegaly	15
Hypotension	20*
Metabolic disturbance	
Hyponatremia	10
Hypoglycemia	10
Hypoxemia	10
Hypercarbia	10*
Decrease in GFR	10*

**Table 4:** Interpretation of the scoring system

Myxedema score	Interpretation
<25	Unlikely
26-59	Suggestive
>60	Highly suggestive

Usually, myxedema coma is improbable, with a score below 25, but a score above 60 strongly indicates the possibility of this diagnosis [1]. It's essential to keep in mind that this score provides suggestions rather than a definitive diagnosis. Nevertheless, utilizing this tool assisted in reinforcing the diagnosis in the case above. Aside from his newly diagnosed untreated hypothyroidism, identifying a distinct precipitating event, in this case, proved to be challenging, resulting in a calculated score of 80. Points were given for his degree of hypothermia, somnolent state, bradycardia, hypotension, hypercarbia, and decrease in GFR. Studies also go further to suggest that higher scores had significantly higher mortality, with myxedema coma score above 110 showing 100% mortality, making this condition a true medical emergency [5]. Therefore it is of utmost importance that once the diagnosis is suspected or identified, treatment is initiated. 

Due to the multi-systemic effects, patients must be admitted to the intensive care unit. To reverse the multi-systemic manifestations of severe hypothyroidism, thyroid hormone replacement (intravenous levothyroxine 200-400mcg) is the mainstay of treatment and must be given immediately. Liothyronine is the body's active hormone and may also be administered, as it provides a quick onset of action compared to levothyroxine; however, its availability is limited, and some reports have associated its use with poor cardiovascular outcomes [4]. T3 was not administered in this case as it was unavailable at the initial institution, and thereafter the patient had a good clinical response with levothyroxine only. 

In addition to hormone replacement, a stress dose of steroids should be given (intravenous hydrocortisone 50-100mg every 6 to 8 hours) due to the possibility of concomitant adrenal insufficiency. Fortunately, there was no adrenal insufficiency, as evidenced by the patient's normal cortisol level, hence avoiding the need for long-term steroid replacement therapy, which has its own set of adverse effects.

One important sign identified here is respiratory failure, the initial presenting feature in this case. This results from myopathy of the respiratory muscles leading to hypoventilation and hypercapnia. The pathogenesis remains unclear but is thought to be related to the role of thyroid hormone in cellular metabolism and Na-K-ATPase activity. Macroglossia, pharyngeal, and vocal cord edema also contribute to airway obstruction leading to hypercapnia and coma [4]. Currently, there is no data that correlates the severity of hypothyroidism with the degree of respiratory failure and whether this myopathy resulting in hypoventilation is completely reversible. In 2014, Fukusumi reported a case of persistent respiratory failure requiring long-term non-invasive ventilation post myxedema coma despite normalization of thyroid function [6]. Further studies are needed to investigate the long-term effects of myxedema coma on physiological function.

From this case above, it is unclear whether his history of myxedema coma led to his decline; however, this condition is well known to have a high mortality rate. Cardiovascular dysfunction, the need for pressor support, and older age were determined to be major factors leading to death in a Japan-based study. Fortunately, the mortality rate has improved from 80% to 20-50%, with in-hospital mortality being 29% due to more intensive management guidelines in these patients [2,7]. Rodríguez et al. also conducted a small prospective study to examine the factors associated with the mortality of patients with myxedema coma. Four of 11 patients died within two weeks of receiving treatment. They found that the degree of consciousness, Glasgow scores, and the severity of illness measured by APACHE II score at presentation were all significant factors leading to mortality [8]. An APACHE II score over 20 is known to be associated with a higher mortality risk. Considering this information, the APACHE II score of the patient above was retrospectively calculated to be 35; this is well in keeping with his poor prognosis. Recognizing these factors enables healthcare professionals to anticipate potential decline in order to administer more aggressive and targeted treatments.

## Conclusions

Overall, myxedema coma is a rare but life-threatening complication of severe hypothyroidism. This case brings awareness to an atypical presentation. Clinicians should maintain a high index of suspicion for myxedema coma, especially in elderly patients presenting with respiratory failure and altered mental status. Improved understanding of the condition and utilization of the myxedema scoring tool to guide prompt initiation of appropriate treatment reduces morbidity and mortality rates significantly.
